# Editorial: Biology of Brain Disorders

**DOI:** 10.3389/fncel.2017.00366

**Published:** 2017-11-21

**Authors:** Daniela Tropea, Andrew Harkin

**Affiliations:** ^1^Neuropsychiatric Genetics, Functional Genomics, Psychiatry, Trinity College, Dublin, Ireland; ^2^Trinity Center for Health Sciences St. James Hospital, Dublin, Ireland; ^3^Trinity College Institute of Neuroscience, Trinity College, Dublin, Ireland; ^4^School of Pharmacy and Pharmaceutical Sciences, Trinity College, Dublin, Ireland

**Keywords:** brain, disorders, neurodegenerative, neuropsychiatric, injury

Brain disorders are a major global healthcare problem. The scale of the problem is growing as the population ages and the numbers of people affected, directly or indirectly, is on the rise. The need for ongoing research to gain a greater understanding of the brain, structure, and function in health and disease, has never been more important. In the past, progress has been made largely by technological advances e.g., the clinico-pathological description of degenerative disorders followed by the discovery of chemical transmitters and introduction of pharmacological treatments. The introduction of analytical methods allowed for the identification of chemical transmitters affected providing insights into phenotypes of cells lost in degenerative diseases. More recent times have heralded the genetic analysis of causation leading to the recognition of disease genes for most brain disorders. The greater significance is that genetic analysis allowed scientists to access the endogenous origins of a disease. The current era of genomics in addition to analysis of the transcriptome, proteome, and metabolome allows for the study of all genes at all stages from initiation to death to provide a more complete picture. Genetic analysis has further allowed for the development of models to understand pathways and improved earlier diagnosis in pre-symptomatic stages. However, no treatment based on genetic analysis has yet to reach clinical utility although ongoing efforts are getting close to finding potential solutions such as amyloid therapies for Alzheimer's disease.

Despite these advances in understanding brain disorders, significant challenges remain. The human brain is the most complicated machine we know. There are numerous cell types including neurons, astrocytes, microglia, and oligodendrocytes including further variations in the types of neurons and glia. There is a specificity for particular regions of the nervous system or cell type in different disorders. Why this is the case and how this comes about remain a mystery and is part of the challenge that scientists face. Furthermore, although there is clearly a genetic component to many brain disorders, it is equally clear that there are environmental risk or attenuating factors. This remains a critical issue in genetic forms of brain disorders when many mutations show incomplete penetrance. To address these complex challenges, there is no one best approach. Reductionism to the level of molecules and cells is inevitable but eventually scientists must return to the human brain at a systems level to determine neuronal networks, regional and whole brain structure and function. There is a reliance on cellular models, cell lines, primary cells, and stem cells and model organisms, from flies to rodents, to gain invasive and fundamental insights not possible in human experiments. There are a myriad of new technologies available heralding a next generation of progress ranging from advances in molecular biology, inducible pluripotent stem cells, genomic editing, optogenetics, and structural and functional MRI. MRI is of particular value as it one of a few tools that may be used both in the clinic and in animal experiments and can therefore provide a translational framework for the combination of model organisms with multimodal imaging and other modalities such as EEG, behavior and post-mortem cell and molecular analysis to elucidate mechanisms underlying imaging phenomena in patients with brain disorders (McIntosh et al.).

This collection of research articles represents a cross sectional sample of current approaches to study brain disorders. Submissions range from scientists working on genetic, epigenetic, and transcriptional aspects, on molecular and cellular mechanisms associated with neuronal plasticity and degeneration, on elucidating a role for brain glia, brain imaging in animals and humans in addition to translational and interventional research in clinical populations. There is a growing consensus that a multi-level approach is required to uncover mechanisms in brain function to provide pathways for the development of new treatments for brain disorders (Carcone and Ruocco).

In some cases genetic analyses across different brain disorders (schizophrenia, depression, anxiety, neurodegenerative disorders) point to common factors (Di Re et al.). In other instances, as revealed by genome wide association studies (GWAS), there are some diseases which are polygenic with multiple genes contributing to cumulative risk (Redenšek et al.). Some mechanisms and factors are common across diseases such as processes leading to synaptic dysfunction indicating that common protective strategies may be used (Criscudo et al.). Another example is given by the growth factor insulin-like growth factor 1 (IGF1) which is a common modulator of neurodevelopmental disorders and aging (Wrigley et al.), or molecular determinants of epigenetic controls: HDAC3 and HDAC4—enzymes controlling histone deacetylation—activate mechanisms for neurodegeneration, cognition, and brain injury (Wu et al.; Yang et al.).

The field of bioenergetics is emerging as common ground in the pathophysiology of several brain disorders: Mathur et al. show that gene pathways linked to carbohydrate metabolism are linked to the severity of multiple sclerosis. Lactate shuttles are viewed as operators for homeostasis in brain trauma and neurodegenerative diseases (Mason).

Brain plasticity, the brain's ability to adapt and capacity to repair itself following injury, is also a critical area of research at present. The rescue of brain tissue following ischemic damage (Alia et al.) and the improvement in function following rehabilitation after stroke (Constans et al.) are representative of this largely untapped area. There is a growing awareness of the importance of plasticity as a central mechanism underlying adaptive change associated with the pathophysiology and treatment of brain disorders.

Insight into mechanisms that underlie the onset and progression of brain disorders may help identify new avenues for therapeutic intervention. Emerging evidence that common deficits are present in several brain disorders point to mechanisms that generalize across disorders. Further progress will no doubt be related to technological advances in molecular screening and detection, the manipulation and study of neuronal circuits, tools for assessment of systems/regional connectivity, and whole brain function which may be used in both animal and human experiments and the analysis of big data drawn from multi-centered strategic partnerships in the development of projects of greater scale.

## Author contributions

All authors listed have made a substantial, direct and intellectual contribution to the work, and approved it for publication.

### Conflict of interest statement

The authors declare that the research was conducted in the absence of any commercial or financial relationships that could be construed as a potential conflict of interest.

